# Trait‐Mediated Competition for Light Underpins Plant Diversity Loss Under Eutrophication

**DOI:** 10.1111/gcb.70521

**Published:** 2025-09-23

**Authors:** Tianyuan Tan, Huamei Xia, Cong He, Yao Wei, Xiang Liu, Zhenhua Zhang, Jin‐Sheng He, Lin Jiang

**Affiliations:** ^1^ State Key Laboratory of Herbage Improvement and Grassland Agro‐Ecosystems, College of Pastoral Agriculture Science and Technology Lanzhou University Lanzhou China; ^2^ Qinghai Haibei National Field Research Station of Alpine Grassland Ecosystems, Northwest Institute of Plateau Biology Chinese Academy of Sciences Xining China; ^3^ Institute of Ecology, College of Urban and Environmental Sciences, Key Laboratory for Earth Surface Processes of the Ministry of Education Peking University Beijing China; ^4^ School of Biological Sciences Georgia Institute of Technology Atlanta Georgia USA

**Keywords:** functional trait, light competition, nutrient enrichment, plant economics spectrum, species diversity, species gain and loss

## Abstract

Eutrophication is a major driver of plant diversity loss, yet the underlying mechanisms remain poorly understood. In particular, the role of eutrophication‐induced light limitation in regulating plant diversity in natural communities has rarely been examined directly. Here we show that experimental light addition to the understory of a natural alpine grassland consistently restored lost diversity under different nutrient enrichment regimes. Under nitrogen enrichment, light addition recovered diversity primarily by promoting species gains, whereas under phosphorus enrichment, it primarily reduced species losses. When both nitrogen and phosphorus were enriched, light addition simultaneously increased species gains and reduced losses. These effects were primarily driven by shifts in the colonization and extinction of species with resource‐acquisitive strategies (i.e., those with high specific leaf area and low leaf dry matter content), emphasizing the critical role of trait‐mediated competition for light in biodiversity loss. Our findings point to light competition as a key driver of eutrophication‐induced plant diversity loss, suggesting that managing light availability could help mitigate these losses in natural ecosystems.

## Graphical abstract

Eutrophication often reduces plant diversity by making the ground layer darker, which reduces understory plants. In a natural alpine grassland, we added LED light beneath the canopy and saw diversity return across different nutrient treatments: with added nitrogen fertilizer, light mainly increased new species; with added phosphorus fertilizer, it mostly reduced species losses; with both nutrients, it did both. These processes were linked to species with common leaf traits, suggesting that managing understory light can help protect biodiversity in nutrient‐enriched ecosystems.

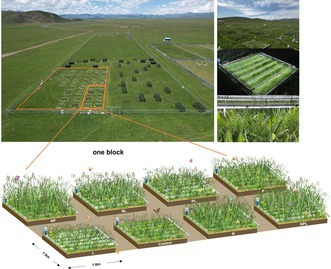

## Abstract (zh)

摘要: 富营养化是植物多样性丧失的主要驱动因素, 但其内在机制尚不明确。此外, 目前尚缺乏针对富营养化所引起的光限制如何调控自然植物群落多样性的直接实验证据。本研究发现在氮添加下, 补光主要通过促进物种获得来恢复多样性; 而在磷添加下, 它主要通过减少物种损失来维持多样性。当氮和磷同时添加时, 补光则同时促进物种获得并减少物种损失。这些效应主要是由具有资源获取型性状(即比叶面积高、叶干物质含量低的物种)的物种的定殖与灭绝变化所驱动的, 这强调了性状介导的光竞争在生物多样性丧失中的关键作用。我们的研究结果表明, 光限制是富营养化导致植物多样性丧失的关键驱动因素, 并提出在天然生态系统中调控光可利用性可能有助于减轻这种损失。

## 1 Introduction

Anthropogenic activities, including intensified fertilizer use and fossil fuel combustion, have profoundly altered nitrogen and phosphorus cycles (Galloway et al. 2004, 2008; Peñuelas et al. 2012, 2013; Smil 2000). These changes increase nitrogen and phosphorus input into terrestrial ecosystems, enhancing plant biomass and highlighting their status as key limiting nutrients for primary production (Du et al. 2020; Elser et al. 2007; Harpole et al. 2016). However, nitrogen and phosphorus enrichment frequently reduce plant diversity (Bobbink et al. 2010; Ceulemans et al. 2014; Clark and Tilman 2008; Elser et al. 2007; Harpole et al. 2016), posing risks to ecosystem function and stability (Hooper et al. 2005; Loreau et al. 2001; Tilman et al. 2014). As human impacts on nitrogen and phosphorus cycles continue to intensify (Canfield et al. 2010; Yuan et al. 2018), understanding mechanisms of this eutrophication‐induced diversity loss is increasingly urgent.

Multiple hypotheses have been proposed to explain plant diversity loss under eutrophication, with the light limitation hypothesis receiving much recent attention. This hypothesis posits that nutrient enrichment increases plant biomass, reducing understory light availability and causing the extinction of species intolerant to low‐light conditions (DeMalach et al. 2017; Newman 1973). Despite the long history of the hypothesis (Newman 1973) and renewed interest among ecologists (DeMalach et al. 2017; Eskelinen et al. 2022; Hautier et al. 2009), the role of light competition in shaping the diversity of eutrophic communities remains contentious (DeMalach and Kadmon 2017; Harpole et al. 2017). A major challenge in testing this hypothesis is the difficulty of manipulating light availability without disturbing the biotic community. Ideally, this would involve adding light to the understory, but such experiments are rare due to logistic constraints (Eskelinen et al. 2022; Hautier et al. 2009), particularly in natural field settings. Notably, the only field study to date investigating light amendment reported inconsistent effects on plant species diversity (Eskelinen et al. 2022). Moreover, it is also unclear whether light limitation would have consistent effects across different types of nutrient enrichment, such as nitrogen versus phosphorous. A robust test of the light limitation hypothesis should also consider alternative hypotheses (Rajaniemi et al. 2003; Dickson and Foster 2011; Eskelinen et al. 2022). For instance, the niche dimension hypothesis suggests that nutrient addition reduces the number of limiting resource niches, weakening tradeoffs that allow species to coexist, thereby contributing to diversity loss (Harpole and Tilman 2007; Harpole et al. 2016). Additionally, changes in soil properties due to eutrophication, such as acidification (Houdijk et al. 1993; van den Berg et al. 2005) and increased toxic metal concentrations (van den Berg et al. 2005; Horswill et al. 2008; Tian et al. 2016), may also drive diversity decline. However, few studies have tested these competing hypotheses simultaneously (Band et al. 2022).

Species functional traits regulate their responses to abiotic and biotic environments (Violle et al. 2007; Violle and Jiang 2009), including patterns of colonization and extinction (Li et al. 2015; Yang et al. 2018, 2019). Partitioning changes in species diversity into species‐level gains (colonization) and losses (extinction) and linking them to functional traits can help elucidate the processes shaping species diversity. This approach is facilitated by the concept of the plant economics spectrum, which positions rapid‐growing, resource‐acquisitive species on one end and slow‐growing, resource‐conservative species on the other (Wright et al. 2004; Reich 2014). For example, fertilization has been shown to reduce plant diversity in tundra meadows by increasing the extinction risk of resource‐conservative species, such as those with low specific leaf area (SLA), without affecting colonization (Kaarlejärvi et al. 2017). This aligns with studies reporting that nutrient enrichment can favor resource‐acquisitive species over conservative ones (Eskelinen et al. 2022; Eskelinen and Harrison 2015; Fujita et al. 2014; Kaarlejärvi et al. 2017; Zhu et al. 2020). If light limitation mainly causes the loss of resource‐conservative species under eutrophication, light addition may be expected to primarily benefit these species (Eskelinen et al. 2022), potentially by altering their colonization and extinction rates. However, it remains largely unknown how light availability influences the gain and loss of species with different resource‐use strategies.

**FIGURE 1:**
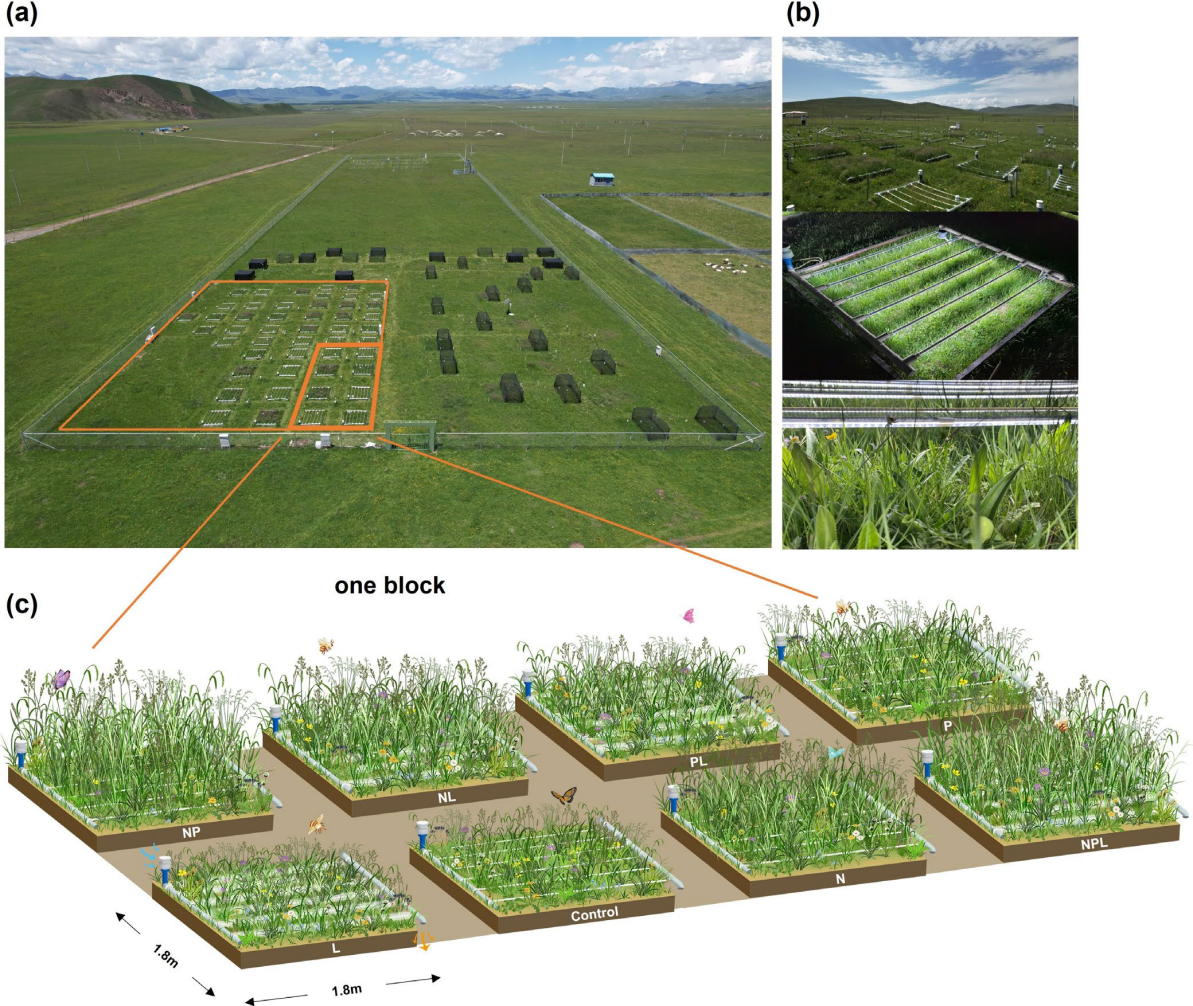
Experimental setup. (a) Landscape view of the light competition experiment in a natural alpine grassland. (b) Photographs illustrating the experiment: The upper panel shows the layout of the experimental plots; the middle and lower panels depict light addition to the understory via LED strips at night and in the daytime, respectively. (c) Overview of the experimental design, highlighting treatment groups and expected treatment effects. Details on the light addition device are provided in Figure S1.

We investigated the role of light limitation in regulating plant diversity using a 3‐year field experiment (2021–2023) in an alpine grassland on the Tibetan Plateau, independently manipulating understory light and soil nitrogen and phosphorus availability. The alpine grassland is characterized by a short, cool summer (June–July, mean temperature 9.9°C) and a long, cold winter (October–March, mean temperature −7.9°C), yet sustains high plant diversity (40–50 species m^−2^). This distinct ecological context provides a unique opportunity to test mechanisms underlying light‐mediated plant diversity loss under eutrophication, which has been previously conducted in temperate grasslands only (Eskelinen et al. 2022). We increased understory light using full‐spectrum LED lamps (Figure 1; Figure S1) and applied nutrients factorially (N, P, and NP) to distinguish potential nutrient‐specific effects of light addition, which have not been explored previously. In addition to light limitation, we also considered altered soil properties and reduced niche dimension as alternative mechanisms driving diversity loss. Our findings point to light limitation as the primary mechanism responsible for the decline in plant diversity following eutrophication. Surprisingly, light addition primarily influenced the extinction and colonization of species with resource‐acquisitive strategies, with these effects varying between nitrogen and phosphorus treatments.

## 2 Materials and Methods

### 2.1 Study Site

Our study site is located in a high‐altitude (3200 m above sea level) alpine grassland at the Qinghai Haibei National Field Research Station of Alpine Grassland Ecosystem (37°29′–37°45′N, 101°12′–101°23′ E) on the Tibetan Plateau. The mean annual temperature is −1.1°C, and mean annual precipitation is 485 mm (Zhang et al. 2021). In this region, the soil is classified as mollisols according to the US Department of Agriculture Soil Taxonomy (Liu et al. 2018). The alpine grassland is dominated by grasses, but forbs make up the majority of species, accounting for approximately 72.5% of the community.

### 2.2 Experiment Design

Our experiment took place in a 25 × 35 m section of a 100 × 200 m fenced area, established in 2018, with experimentation starting in 2021. We manipulated three key resources, including understory light, soil nitrogen, and phosphorus, in a randomized block design. There were eight treatment combinations, crossing two light levels (ambient vs. light addition), two nitrogen levels (ambient vs. 10 g/m^2^ N added as NH_4_NO_3_), and two phosphorus levels (ambient vs. 5 g/m^2^ P added as Ca(H_2_PO_4_)_2_). Each combination had six replicates (blocks), totaling 48 plots (1.8 × 1.8 m each). One plot intended for N fertilization was accidentally treated with both N and P in 2021, and intentionally so afterwards; this plot was classified as an NP plot.

Light addition was implemented by suspending six parallel full‐spectrum LED light strips at ~12 cm above the ground within each plot (Figure 1; Figure S1). Compared to conventional LED lights, full‐spectrum LED lights more accurately replicated the spectral profile of natural sunlight (Figure S1b). From May to September, the LED strips in the light‐amended plots were powered on from 8:00 to 18:00. In the ambient‐light plots, the same LED strips were installed and powered on, but were wrapped in aluminum foil to prevent light exposure, as a way to control for any unintended physical or microenvironmental effects of the LED strips. N and P were added to the experimental plots annually (mid‐May) from 2021 to 2023.

### 2.3 Data Collection

From 2020 to 2023, we recorded all plant species within two permanent 50 × 50 cm quadrats in each plot in late August, when plant communities reached peak biomass. Within each plot, a 15 × 60 cm strip was randomly selected and clipped at ground level. Plant samples were sorted by species, oven‐dried at 65°C for 48 h, and weighed to estimate biomass. In August 2023, we measured five commonly used plant traits—height, leaf dry matter content (LDMC), specific leaf area (SLA), leaf area, and leaf nitrogen content (leaf N)—to assess plant resource acquisition strategies (Díaz et al. 2016). Most trait data were collected from the control plots, but for a few rare species, we gathered data from a nearby area outside the experimental plots. Trait measurements followed standard protocols (Pérez‐Harguindeguy et al. 2013).

In mid‐August 2023, photosynthetically active radiation (PAR) in the understory of each plot was measured using a Sunfleck PAR Ceptometer (AccuPAR LP‐80, Decagon Devices, Pullman, WA, USA). To quantify PAR under both natural conditions and light amendment, measurements were conducted in two steps. First, ambient PAR was measured at 10 cm above the surface with the LED lights off, recorded at noon over four consecutive cloudless days. Second, PAR emitted from the LED strips was measured at the same height (10 cm) during nighttime.

In late August 2023, we randomly collected three soil cores (3.5 cm in diameter, 10 cm in depth, and 40 cm apart) from each plot and combined them into a composite soil sample for analysis of soil properties. Plant roots and debris were removed, and the remaining soil was passed through a 2 mm sieve. Soil pH was measured in a 1:2.5 soil‐to‐water suspension using a pH meter (FE28, Mettler‐Toledo, Greifensee, Switzerland). Soil ${\mathrm{NO}}_{3}^{-}$ and ${\mathrm{NH}}_{4}^{+}$ concentrations were determined with a Fully Automatic Kjeldahl Nitrogen Analyzer (K1160, Jinan Hanon Instruments Co. Ltd., Jinan, China). Soil available P was determined using a UV–Vis spectrophotometer (UV‐1800PC, Shimadzu, Kyoto, Japan). Water‐soluble salts, including aluminum (Al^3+^), iron (Fe^3+^) and manganese (Mn^2+^), were analyzed using an inductively coupled plasma optical emission spectrometer (iCAP‐7200, Thermo Fisher Scientific, Waltham, MA, USA).

Air temperature and humidity in each plot were monitored using HOBO H21‐USB data loggers (Onset Computer Cooperation, Bourne, MA, USA), installed 15 cm above the ground. Data were collected every 30 min between May 2021 and August 2023.

### 2.4 Statistical Analysis

All statistical analyses were performed in R 4.2.2 (R Core Team 2022). Species richness for each plot was determined by counting the number of species present in the two 50 × 50 cm quadrats. The effects of experimental treatments on species richness, total community biomass, functional group biomass, PAR, and soil physicochemical properties were analyzed using data from the final year of the experiment (2023), when treatment effects were most pronounced. To quantify species gains and losses in each plot, we compared plant species composition within the plot between the pre‐treatment year (2020) and the final experimental year (2023), with gains estimated as the number of species absent in 2020 but present in 2023, and losses as the number of species present in 2020 but absent in 2023. To assess potential baseline differences among treatment plots, we examined initial species richness and species‐level community composition in 2020. Differences in species richness were analyzed using linear mixed‐effects models (LMMs), while differences in community composition, based on Bray–Curtis dissimilarities, were examined using permutational multivariate analysis of variance (PERMANOVA) with 999 permutations. Neither analysis detected significant differences among treatment plots at the start of the experiment (see Figure S2).

We used linear mixed‐effects models (LMMs) to assess the effects of light addition (L), N addition (N), and P addition (P) on the biomass of community and functional groups, species richness, the number of species gains/losses, and PAR and soil properties. We also used random forest models to analyze the relative importance of different variables in explaining species richness and the number of species gains/losses across all treatments (Liaw and Wiener 2007). These variables include light availability, niche dimension (the number of added resources), soil metal concentrations (represented by the first principal component of principal component analysis of soil Al, Fe, and Mn concentrations), as well as microclimate (represented by the first principal component of principal component analysis of air temperature and relative humidity).

We conducted a principal component analysis (PCA) on the five functional traits to reduce dimensionality and quantify differences in resource‐use strategies among species. The first principal component (PC1), which explained 44.51% of total trait variation, captured a gradient from tall height and large LDMC—traits associated with resource‐conservative strategies—towards high SLA and leaf N content, traits linked to more acquisitive strategies. The second principal component (PC2), explaining 22.7% of total trait variation, captured a gradient from small to large leaf area.

To assess the effects of functional traits on species gain and loss probabilities, we performed logistic regressions of species gain and loss as functions of their traits, including height, SLA, leaf N content, LDMC, and leaf area, by using generalized linear mixed‐effects models (GLMMs). Species gain and loss events were treated as binary response variables (1: gain/loss; 0: no gain/loss). Model selection was used to identify the significant traits included in the best‐fitting model (based on Akaike's information criterion). To assess multicollinearity, we examined pairwise Pearson correlation coefficients among the five plant traits and found all |*r*| < 0.6 (see Figure S3). We further calculated variance inflation factors (VIF) for the fixed effects in the GLMMs; all VIF values were below 3 (maximum = 2.72), well under the commonly accepted threshold of 5 (see Table S1).

LMMs were implemented using the “lmer” function in the lme4 package (Bates et al. 2015), with block included as a random factor. Community composition differences among treatments were evaluated using PERMANOVA with the “adonis2” function in the vegan package (Oksanen et al. 2022). Differences in richness and species gain/loss among treatments were assessed using the emmeans package (Lenth 2023). GLMMs were carried out using the “glmer” function from the lme4 package (Bates et al. 2015), and model selection was performed with the “dredge” function from the MuMIn package (Bartoń 2022). Phylogenetic signal was tested using the “phylosig” function in the phytools package (Revell 2012).

## 3 Results

### 3.1 Treatments Effects on Abiotic Properties

As expected, nitrogen (N) addition increased soil inorganic N content, with ${\mathrm{NO}}_{3}^{-}$ and ${\mathrm{NH}}_{4}^{+}$ concentrations rising by 88.0% and 109.3%, respectively (Figure S4 and Table S2). Nutrient addition, however, did not affect soil pH (Figure S4; Table S2). On the other hand, soil Al^3+^ and Fe^3+^ concentration, but not Mn^2+^ concentration, declined following nutrient amendment (Figure S4; Table S2). Understory light availability declined consistently under nutrient amendment, by 37.17%, 56.52%, and 92.59% in N, P and NP treatments, respectively (Figure S5 and Table S3). Light addition increased understory light intensity, but did not influence soil properties or microclimatic variables (Figures S4–S6; Tables S2 and S3).

### 3.2 Treatments Effects on Biomass and Species Richness

N, P, and NP increased community biomass by an average of 28.1%, 26.8%, and 142.1%, respectively (Figure S7a and Table S4), driven predominantly by grasses (Figure S7b; Table S4). While other plant functional groups (forbs, sedges, and legumes) showed varying responses (Figure S7c–e; Table S4), the biomass of forbs—the most species‐rich group—remained largely unaffected by nutrient amendment (Figure S7c; Table S4).

**FIGURE 2:**
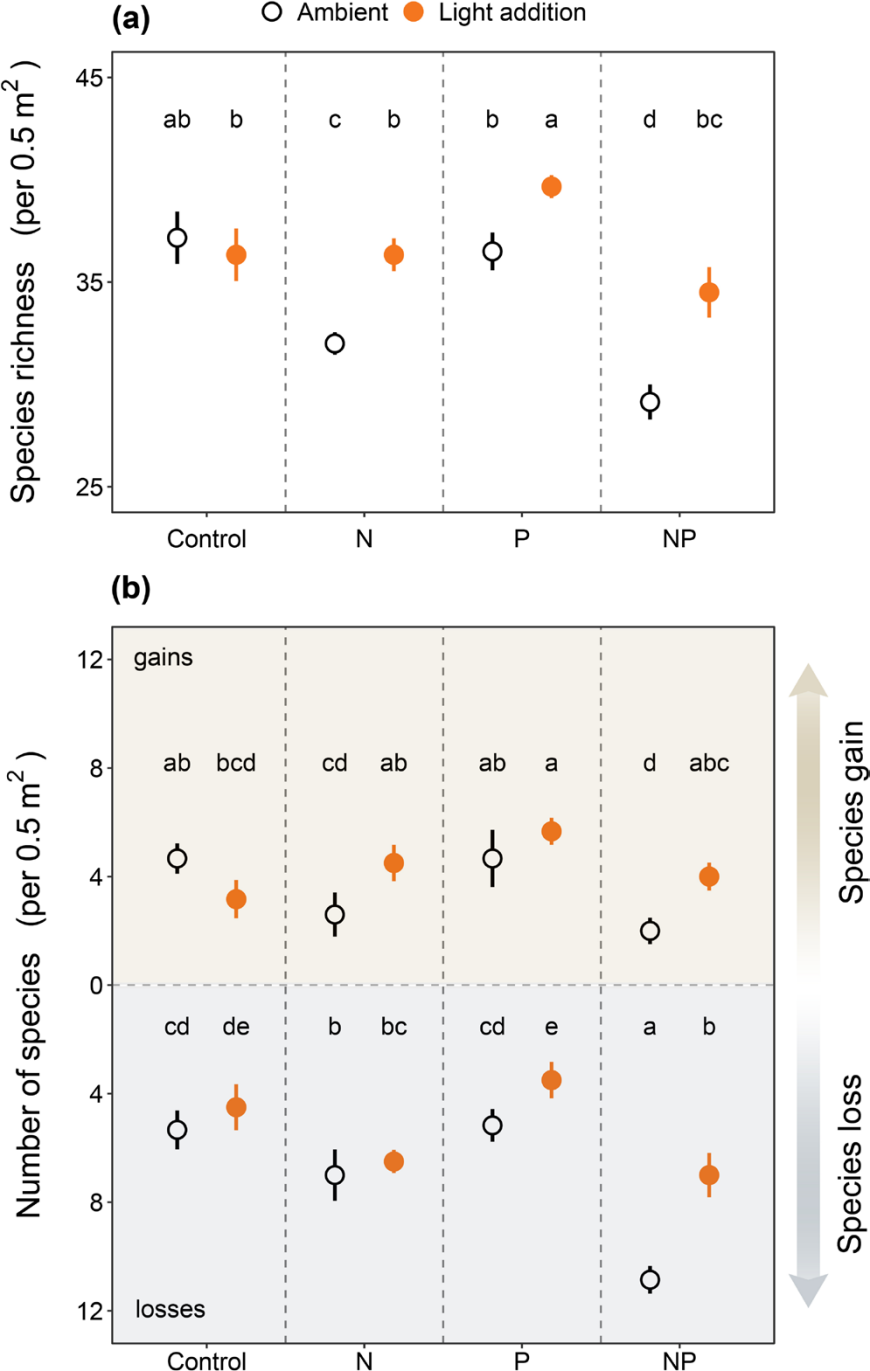
Effects of experimental treatments on plant species richness, gains and losses. The effects of light addition (L), N addition (N) and P addition (P) on plant species richness at the end of the 3‐year experiment (i.e., in 2023) (a) and the number of species gains and losses between 2023 and 2020 (b). Data are presented as mean + s.e.m. Different letters denote significant differences between treatment means (*p* < 0.05), conducted separately for species gain and loss.

By the end of the 3‐year experiment, nitrogen addition alone reduced species richness by an average of 5.2 species, while P addition alone had no discernible effect on plant richness (Figure 2a; Table S5). The combination of N and P resulted in the greatest diversity losses, with an average reduction of 8.0 species (Figure 2a; Table S5), indicating the synergistic negative impact of these nutrients. Notably, while adding light had no effect on species richness under ambient‐nutrient conditions, it consistently restored lost diversity across all nutrient‐enriched treatments (Figure 2a; Table S5).

### 3.3 Trait‐Dependent Species Colonization and Extinction

The changes in species richness under N and NP enrichment reflected both reduced species colonization and increased extinction rates (Figure 2b; Table S5). We found that light addition countered these losses through different pathways depending on the nutrient regime. Under N enrichment, where diversity declined mainly due to reduced species gains, supplemental light primarily promoted colonization. Under P enrichment, where losses dominated, added light reduced extinctions. Under NP enrichment, light addition simultaneously increased gains and reduced losses, fully restoring diversity (Figure 2b; Table S5).

**FIGURE 3:**
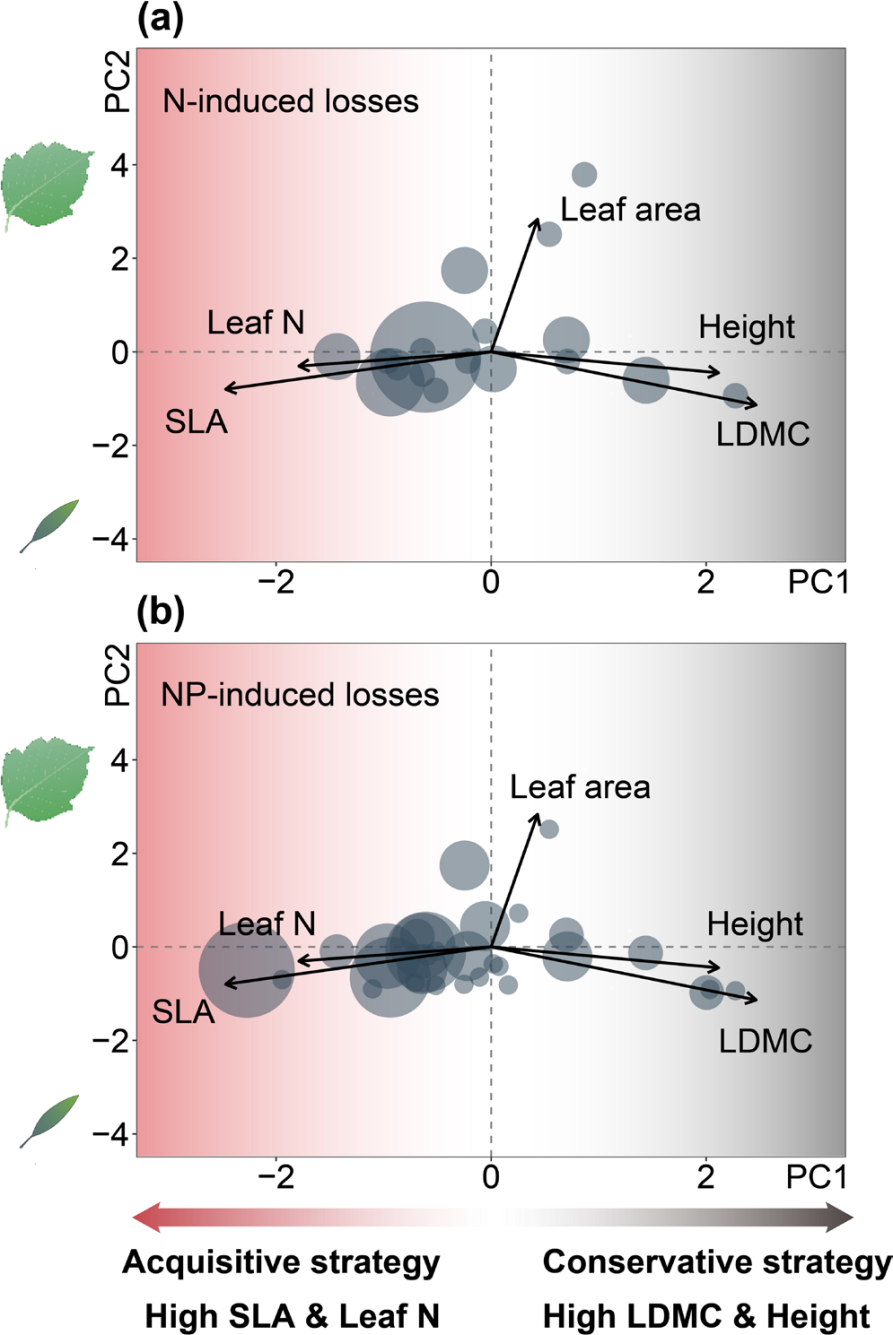
Traits of species lost under eutrophication. Principal component analysis of plant traits from control plots, highlighting traits of species that were lost after N (a) and NP (b) addition. Species losses were primarily observed among resource‐acquisitive species. Each circle represents a species that was lost, with the circle size proportional to the number of extinctions observed. Axis labels show each principal component (PC1, 44.51% of total trait variation explained; PC2, 22.7% of total trait variation explained). Leaf graphics by T. Saxby and L. Heydon (lntegration and Application Network, University of Maryland Center for Environmental Science, http://ian.umces.edu/imagelibrary/).

**FIGURE 4:**
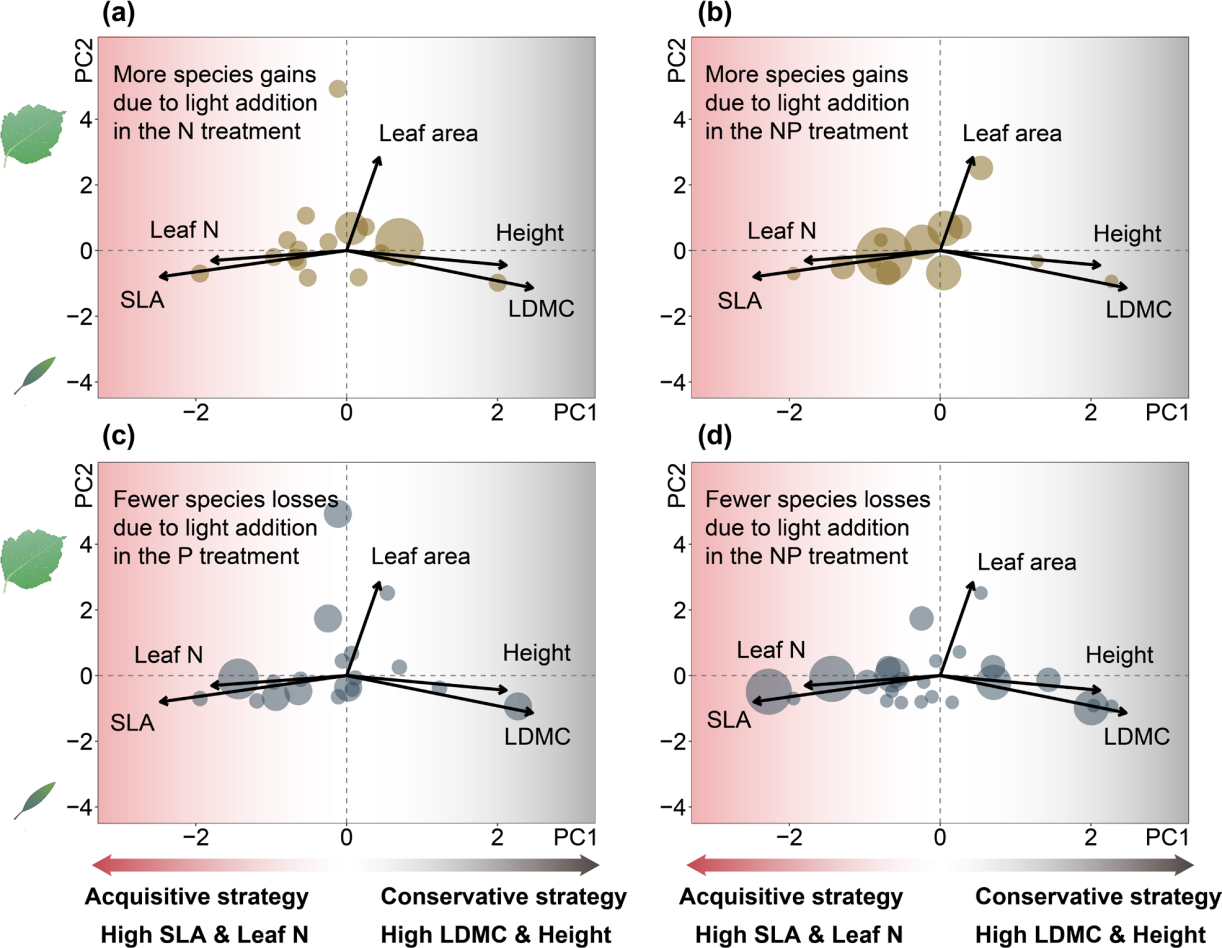
Traits of species gained or rescued from loss after light addition. Principal component analysis of plant traits from control plots, highlighting traits of species gained only after light addition in N‐amended (a) and NP‐amended plots (b), as well as traits of species rescued from loss by light addition in P‐amended (c) and to the NP‐amended plots (d). Both gaines and rescues were primarily observed among resource‐acquisitive species. Each circle represents a species gained or rescued, with the circle size proportional to the number of extinctions observed. Axis labels show each principal component (PC1, 44.51% of total trait variation explained; PC2, 22.7% of total trait variation explained). Leaf graphics by T. Saxby and L. Heydon (lntegration and Application Network, University of Maryland Center for Environmental Science, http://ian.umces.edu/imagelibrary/).

These turnover patterns were largely trait‐dependent. Species gained or lost under different nutrient and light conditions were predominantly resource‐acquisitive. These species, characterized by higher specific leaf area (SLA) and leaf nitrogen content but lower leaf dry matter content (LDMC), were mostly forbs (e.g., *Gentiana aristata*, *Gentianopsis paludosa*, *Lomatogonium carinthiacum*) and faced elevated extinction risks in the N and NP treatments (Figure 3). Importantly, the beneficial effects of light addition—whether promoting species gains or preventing losses—were also largely confined to these acquisitive species (Figure 4).

### 3.4 The Role of Different Mechanisms

**FIGURE 5:**
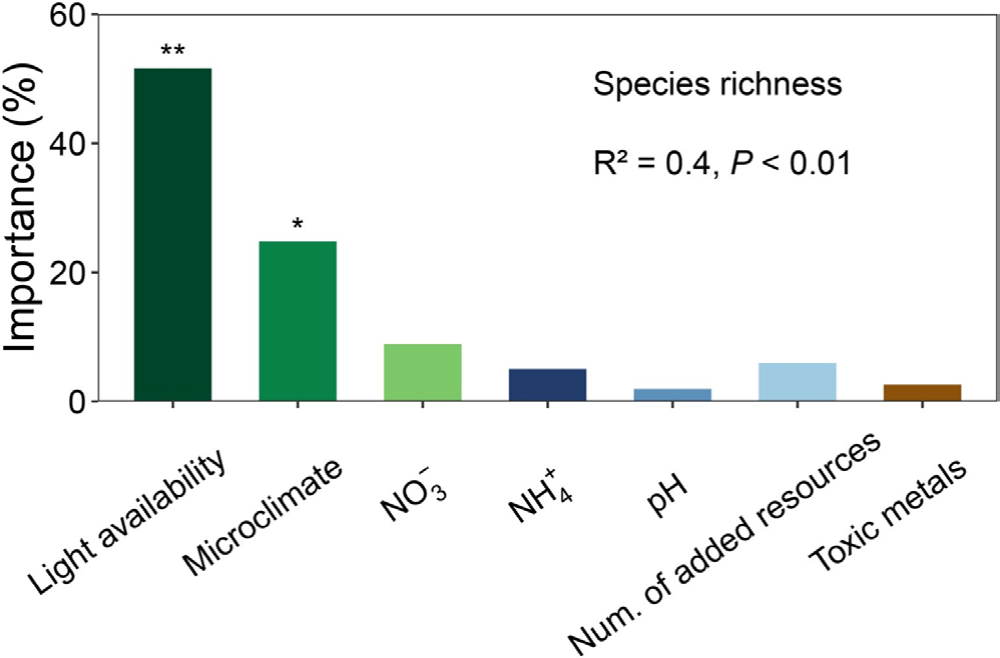
The results of the random forest model for predictors of species richness. Light availability emerged as the best predictor of species richness, surpassing microclimate, soil nitrogen, soil pH, the number of added resources and soil metal concentration. Significant levels: *p < 0.05 and **p < 0.01.

Random forest analysis confirmed understory light availability as the main predictor of species gain and loss (Figure S8) and thus changes in diversity across all treatments (Figure 5). Soil pH, toxic metal concentration, and the number of added resources (representing niche dimensions) were not significant predictors of species gain or loss (Figure S8).

## 4 Discussion

Our experiment yielded two novel, significant findings. First, light addition consistently restored lost plant diversity across all nutrient treatments, whereas changes in niche dimensions or soil properties failed to explain diversity loss under nutrient enrichment. Second, light addition primarily affected the dynamics of resource‐acquisitive species, driving gains and mitigating losses, whereas conservative ones were less affected. These findings provide strong support for the light limitation hypothesis, highlighting the critical role of light availability in maintaining plant diversity in nutrient‐enriched ecosystems.

### 4.1 Nutrient Enrichment Reduces Plant Diversity

Nutrient addition in grasslands is known to increase plant biomass but reduce plant species diversity (Bobbink et al. 2010; Ceulemans et al. 2014; Clark and Tilman 2008; Elser et al. 2007; Harpole et al. 2016). In our study, nutrient enrichment similarly resulted in increased biomass and reduced diversity, particularly in the N and NP treatments. This reduction of plant diversity was driven by reduced species gains and increased species losses, as seen in several other fertilization experiments (Hautier et al. 2009; Kaarlejärvi et al. 2017). The greatest species loss occurred under the combined N and P addition, reflecting a strong synergistic effect between the two nutrients. This synergistic effect may be explained by our study system being co‐limited by N and P, where adding N and P together resulted in greater biomass production than adding either nutrient alone (Figure S7). This, in turn, led to the largest reduction in understory light availability (Figure S5) and, eventually, the largest declines in species diversity in the NP treatment (Figure 2).

Our findings, however, differ from those of previous studies in a key aspect. We found that resource‐acquisitive species had higher extinction risks and lower colonization rates under nutrient enrichment. In contrast, previous studies have reported that resource‐conservative species were more prone to extinction after eutrophication (Eskelinen et al. 2022; Eskelinen and Harrison 2015; Fujita et al. 2014; Kaarlejärvi et al. 2017; Zhu et al. 2020), likely due to their shorter stature (Eskelinen et al. 2022; Eskelinen and Harrison 2015; Kaarlejärvi et al. 2017), which limits their competitive ability for light in productive environments. Our study grassland is dominated by grasses with conservative traits (Figures S7 and S9), which responded positively in biomass to nutrient enrichment. This likely imposed stronger competition on shorter, resource‐acquisitive forbs, which faced greater extinction risks. Note that while plant resource‐use strategies and height are correlated, LDMC, not height, was a significant predictor of species extinction under eutrophication in our study (Table S6), suggesting that acquisitive strategies are more important drivers of species losses. Our findings thus provide a mechanistic, trait‐based explanation for the commonly observed trend that nitrogen addition often favors grasses over forbs (DeMalach et al. 2017; Xia and Wan 2008). Overall, the divergent results between our study and previous studies (Eskelinen et al. 2022; Eskelinen and Harrison 2015; Fujita et al. 2014; Kaarlejärvi et al. 2017; Zhu et al. 2020) reporting eutrophication‐induced loss of conservative species suggest that the trait profiles of dominant species may critically determine community responses to nutrient enrichment.

### 4.2 Light Limitation Underlies Eutrophication‐Induced Diversity Loss

Although niche dimension reductions and changes in soil properties are often suggested as potential mechanisms for fertilization‐driven diversity loss, they played a minimal role in our study (Figure 5; Figure S8). The niche dimension hypothesis (Harpole et al. 2016; Harpole and Tilman 2007), which suggests that resource addition leads to species loss by reducing the number of niche axes of limiting resources and weakening coexistence, was not supported. In fact, adding light consistently reduced, rather than exacerbated, diversity loss caused by nutrient enrichment. Soil pH remained stable across treatments, likely due to the high buffering capacity of the soils (Ng et al. 2022), and no increases in soil metal concentrations were detected (Figure S4; Table S2). These results suggest that neither soil pH shifts nor metal toxicity played a significant role in the observed diversity loss, providing little support for the soil pH‐toxicity hypothesis (Horswill et al. 2008; Houdijk et al. 1993; Tian et al. 2016; van den Berg et al. 2005).

In contrast, our results strongly support the light limitation hypothesis. Consistent with the hypothesis, we found that understory light supplementation restored lost species diversity across multiple nutrient amendment treatments. Two studies, one in greenhouse conditions (Hautier et al. 2009) and another in natural settings (Eskelinen et al. 2022), provided evidence that understory light addition can enhance plant richness in grasslands amended with NPK fertilizers. Building on this foundation, our study is the first, to our knowledge, to explicitly test the light limitation hypothesis across different individual nutrient enrichments (N, P, and NP). By doing so, our study offers novel insights into how specific nutrient additions interact with light availability to shape plant diversity. Under N enrichment, light addition restored diversity by primarily promoting species gains. This effect was driven by resource‐acquisitive species, particularly members of the Asteraceae and Ranunculaceae families. Conversely, under P enrichment, light addition elevated diversity by reducing species losses. This effect was also driven by resource‐acquisitive species, particularly those from the Gentianaceae family. By contrast, Poaceae species remained largely unaffected by nutrient enrichment, whereas Fabaceae species exhibited the greatest losses under NP addition. For the remaining families (≤ 3 species each), diversity responses showed no consistent pattern, likely due to the limited number of species per family. To explore whether the observed patterns of species turnover were phylogenetically structured, we quantified the phylogenetic signal of treatment‐induced species gains and losses using Blomberg et al. (2003). No significant signal was detected (see Table S7), suggesting that phylogenetic relatedness does not account for these patterns.

The contrasting results on species gains and losses in the N and P treatments likely reflect different roles of light limitation under the two types of nutrient enrichment. In N enriched plots, light addition likely promoted species gains by enhancing seed germination in light‐limited environments, as many grassland species, particularly resource‐acquisitive species with small seeds, rely on light for germination (Fenner and Thompson 2006). In P enriched plots, where light availability was less constrained compared to N enriched plots (Figure S5), light supplementation likely mitigated species losses by balancing the light‐to‐nutrient ratio, promoting coexistence in accordance with resource competition theory (Tilman 1982; Tilman et al. 2014). Both mechanisms may have operated under the combined NP enrichment, where light addition both increased species gains and reduced species losses. Overall, light limitation constrained diversity across all nutrient treatments, pointing to the critical role of light availability for regulating plant diversity.

Several caveats of our study should be acknowledged. First, our findings were obtained from alpine grasslands with distinctive habitat conditions. The mechanisms identified—namely, the modulation of colonization–extinction dynamics through trait‐mediated light competition—will need to be reassessed in other ecosystems. Second, our study did not address how light availability influences species' growth patterns and other physiological responses. These responses, which determine how plants persist and perform under resource‐limited and ‐enriched conditions and are therefore central to explaining resource‐induced shifts in community composition and diversity, warrant investigation in future work. Third, besides nutrients and light, other global change drivers, such as climate change and land‐use modification, may also influence plant communities. For example, changes in water availability are known to alter plant diversity and community structure (Yang et al. 2018; Li et al. 2025). Future research should aim to understand whether light competition remains important under altered precipitation regimes and other global change scenarios. Finally, our experiment only lasted 3 years, and therefore we cannot exclude the possibility that our findings may be potentially influenced by transient dynamics (Hastings et al. 2018). Community responses to changes in resource availability may involve lagged demographic processes, slow trait filtering, or feedbacks through nutrient cycling, which can only be detected over longer timescales (e.g., Reich et al. 2018). Experiments with longer durations will therefore be essential to assess whether the observed effects on plant diversity and community assembly are sustained over time.

Eutrophication‐driven losses of plant diversity have significant implications for grassland ecosystem services, including carbon sequestration and livestock production (Tilman et al. 1997). Our results highlight the potential of light management strategies to mitigate such losses. Practices that enhance understory light, such as maintaining herbivory pressure (Borer et al. 2014; Eskelinen et al. 2022) or periodically removing biomass through mowing (Tälle et al. 2016), could help sustain diverse plant communities even as nutrient inputs rise. By demonstrating that light competition is a key mechanism regulating biodiversity under eutrophication in natural communities, and that species with acquisitive growth strategies are particularly sensitive to light limitation, our findings offer practical avenues for conservation and management.

## Author Contributions

**Tianyuan Tan:** data curation, formal analysis, investigation, supervision, visualization, writing – original draft, writing – review and editing. **Huamei Xia:** data curation, investigation, writing – review and editing. **Cong He:** data curation, investigation, writing – review and editing. **Yao Wei:** formal analysis, methodology, visualization, writing – review and editing. **Xiang Liu:** formal analysis, methodology, writing – review and editing. **Zhenhua Zhang:** resources, supervision, writing – review and editing. **Jin‐Sheng He:** conceptualization, funding acquisition, project administration, resources, supervision, validation, writing – review and editing. **Lin Jiang:** conceptualization, funding acquisition, project administration, supervision, validation, writing – original draft, writing – review and editing.

## Conflicts of Interest

The authors declare no conflicts of interest.

## Supporting information


Data S1.

## Data Availability

The data that support the findings of this study are openly available in Figshare at https://doi.org/10.6084/m9.figshare.29019152.

## References

[gcb70521-bib-0001] Band, N. , R. Kadmon , M. Mandel , and N. DeMalach . 2022. “Assessing the Roles of Nitrogen, Biomass, and Niche Dimensionality as Drivers of Species Loss in Grassland Communities.” Proceedings of the National Academy of Sciences 119, no. 10: e2112010119. 10.1073/pnas.2112010119. PMC8915794 35235460

[gcb70521-bib-0002] Bartoń, K. 2022. “MUMIn: Multi‐Model Inference.” R package version 1.47.1. https://CRAN.R‐project.org/package=MuMIn.

[gcb70521-bib-0003] Bates, D. , M. Mächler , B. Bolker , and S. Walker . 2015. “Fitting Linear Mixed‐Effects Models Using lme4.” Journal of Statistical Software 67: 1–48. 10.18637/jss.v067.i01.

[gcb70521-bib-0004] Blomberg, S. P. , T. Garland , and A. R. Ives . 2003. “Testing for Phylogenetic Signal in Comparative Data: Behavioral Traits Are More Labile.” Evolution 57: 717–745. 10.1111/j.0014-3820.2003.tb00285.x. 12778543

[gcb70521-bib-0005] Bobbink, R. , K. Hicks , J. Galloway , et al. 2010. “Global Assessment of Nitrogen Deposition Effects on Terrestrial Plant Diversity: A Synthesis.” Ecological Applications 20: 30–59. 10.1890/08-1140.1. 20349829

[gcb70521-bib-0006] Borer, E. T. , E. W. Seabloom , D. S. Gruner , et al. 2014. “Herbivores and Nutrients Control Grassland Plant Diversity via Light Limitation.” Nature 508: 517–520. 10.1038/nature13144. 24670649

[gcb70521-bib-0007] Canfield, D. E. , A. N. Glazer , and P. G. Falkowski . 2010. “The Evolution and Future of Earth's Nitrogen Cycle.” Science 330: 192–196. 10.1126/science.1186120. 20929768

[gcb70521-bib-0008] Ceulemans, T. , C. J. Stevens , L. Duchateau , et al. 2014. “Soil Phosphorus Constrains Biodiversity Across European Grasslands.” Global Change Biology 20: 3814–3822. 10.1111/gcb.12650. 24895112

[gcb70521-bib-0009] Clark, C. M. , and D. Tilman . 2008. “Loss of Plant Species After Chronic Low‐Level Nitrogen Deposition to Prairie Grasslands.” Nature 451: 712–715. 10.1038/nature06503. 18256670

[gcb70521-bib-0064] DeMalach, N. , and R. Kadmon . 2017. “Light Competition Explains Diversity Decline Better than Niche Dimensionality.” Functional Ecology 31: 1834–1838. 10.1111/1365-2435.12841.

[gcb70521-bib-0010] DeMalach, N. , E. Zaady , and R. Kadmon . 2017. “Light Asymmetry Explains the Effect of Nutrient Enrichment on Grassland Diversity.” Ecology Letters 20: 60–69. 10.1111/ele.12706. 27933739

[gcb70521-bib-0011] Díaz, S. , J. Kattge , J. H. C. Cornelissen , et al. 2016. “The Global Spectrum of Plant Form and Function.” Nature 529: 167–171. 10.1038/nature16489. 26700811

[gcb70521-bib-0012] Dickson, T. L. , and B. L. Foster . 2011. “Fertilization Decreases Plant Biodiversity Even When Light Is Not Limiting.” Ecology Letters 14: 380–388. 10.1111/j.1461-0248.2011.01599.x. 21332902

[gcb70521-bib-0013] Du, E. , C. Terrer , A. F. A. Pellegrini , et al. 2020. “Global Patterns of Terrestrial Nitrogen and Phosphorus Limitation.” Nature Geoscience 13: 221–226. 10.1038/s41561-019-0530-4.

[gcb70521-bib-0014] Elser, J. J. , M. E. S. Bracken , E. E. Cleland , et al. 2007. “Global Analysis of Nitrogen and Phosphorus Limitation of Primary Producers in Freshwater, Marine and Terrestrial Ecosystems.” Ecology Letters 10: 1135–1142. 10.1111/j.1461-0248.2007.01113.x. 17922835

[gcb70521-bib-0015] Eskelinen, A. , W. S. Harpole , M.‐T. Jessen , R. Virtanen , and Y. Hautier . 2022. “Light Competition Drives Herbivore and Nutrient Effects on Plant Diversity.” Nature 611: 301–305. 10.1038/s41586-022-05383-9. 36323777 PMC9646529

[gcb70521-bib-0016] Eskelinen, A. , and S. P. Harrison . 2015. “Resource Colimitation Governs Plant Community Responses to Altered Precipitation.” Proceedings of the National Academy of Sciences 112: 13009–13014. 10.1073/pnas.1508170112. PMC4620885 26438856

[gcb70521-bib-0017] Fenner, M. , and K. Thompson . 2006. The Ecology of Seeds. Cambridge University Press.

[gcb70521-bib-0018] Fujita, Y. , H. O. Venterink , P. M. Van Bodegom , et al. 2014. “Low Investment in Sexual Reproduction Threatens Plants Adapted to Phosphorus Limitation.” Nature 505: 82–86. 10.1038/nature12733. 24240278

[gcb70521-bib-0019] Galloway, J. N. , F. J. Dentener , D. G. Capone , et al. 2004. “Nitrogen Cycles: Past, Present, and Future.” Biogeochemistry 70: 153–226. 10.1007/s10533-004-0370-0.

[gcb70521-bib-0020] Galloway, J. N. , A. R. Townsend , J. W. Erisman , et al. 2008. “Transformation of the Nitrogen Cycle: Recent Trends, Questions, and Potential Solutions.” Science 320: 889–892. 10.1126/science.1136674. 18487183

[gcb70521-bib-0021] Harpole, W. S. , L. L. Sullivan , E. M. Lind , et al. 2016. “Addition of Multiple Limiting Resources Reduces Grassland Diversity.” Nature 537: 93–96. 10.1038/nature19324. 27556951

[gcb70521-bib-0065] Harpole, W. S. , L. L. Sullivan , E. M. Lind , et al. 2017. “Out of the Shadows: Multiple Nutrient Limitations Drive Relationships Among Biomass, Light and Plant Diversity.” Functional Ecology 31: 1839–1846. 10.1111/1365-2435.12967.

[gcb70521-bib-0022] Harpole, W. S. , and D. Tilman . 2007. “Grassland Species Loss Resulting From Reduced Niche Dimension.” Nature 446: 791–793. 10.1038/nature05684. 17384633

[gcb70521-bib-0023] Hastings, A. , K. C. Abbott , K. Cuddington , et al. 2018. “Transient Phenomena in Ecology.” Science 361, no. 6406: eaat6412. 10.1126/science.aat6412. 30190378

[gcb70521-bib-0024] Hautier, Y. , P. A. Niklaus , and A. Hector . 2009. “Competition for Light Causes Plant Biodiversity Loss After Eutrophication.” Science 324: 636–638. 10.1126/science.1169640. 19407202

[gcb70521-bib-0025] Hooper, D. U. , F. S. Chapin , J. J. Ewel , et al. 2005. “Effects of Biodiversity on Ecosystem Functioning: A Consensus of Current Knowledge.” Ecological Monographs 75: 3–35. 10.1890/04-0922.

[gcb70521-bib-0026] Horswill, P. , O. O'Sullivan , G. K. Phoenix , J. A. Lee , and J. R. Leake . 2008. “Base Cation Depletion, Eutrophication and Acidification of Species‐Rich Grasslands in Response to Long‐Term Simulated Nitrogen Deposition.” Environmental Pollution 155: 336–349. 10.1016/j.envpol.2007.11.006. 18164110

[gcb70521-bib-0027] Houdijk, A. L. F. M. , P. J. M. Verbeek , H. F. G. van Dijk , and J. G. M. Roelofs . 1993. “Distribution and Decline of Endangered Herbaceous Heathland Species in Relation to the Chemical Composition of the Soil.” Plant and Soil 148: 137–143. 10.1007/BF02185393.

[gcb70521-bib-0028] Kaarlejärvi, E. , A. Eskelinen , and J. Olofsson . 2017. “Herbivores Rescue Diversity in Warming Tundra by Modulating Trait‐Dependent Species Losses and Gains.” Nature Communications 8: 419. 10.1038/s41467-017-00554-z. PMC5583392 28871154

[gcb70521-bib-0029] Lenth, R. V. 2023. “Emmeans: Estimated Marginal Means, Aka Least‐Squares Means.” R package version 1.8.4. https://CRAN.R‐project.org/package=emmeans.

[gcb70521-bib-0030] Li, H. , J. Peñuelas , S. L. Collins , et al. 2025. “Water Limitation as a Driver of Species Richness Decline in Global Grasslands Under Nutrient Addition.” Plant and Soil 512: 1–10. 10.1007/s11104-025-07253-5.

[gcb70521-bib-0031] Li, S. , M. W. Cadotte , S. J. Meiners , Z. Hua , L. Jiang , and W. Shu . 2015. “Species Colonisation, Not Competitive Exclusion, Drives Community Overdispersion Over Long‐Term Succession.” Ecology Letters 18: 964–973. 10.1111/ele.12476. 26189648

[gcb70521-bib-0032] Liaw, A. , and M. C. Wiener . 2007. “Classification and Regression by RandomForest.” R News 2: 18–22.

[gcb70521-bib-0033] Liu, H. , Z. Mi , L. Lin , et al. 2018. “Shifting Plant Species Composition in Response to Climate Change Stabilizes Grassland Primary Production.” Proceedings of the National Academy of Sciences of the United States of America 115: 4051–4056. 10.1073/pnas.170029911. 29666319 PMC5910805

[gcb70521-bib-0034] Loreau, M. , S. Naeem , P. Inchausti , et al. 2001. “Biodiversity and Ecosystem Functioning: Current Knowledge and Future Challenges.” Science 294: 804–808. 10.1126/science.1064088. 11679658

[gcb70521-bib-0035] Newman, E. I. 1973. “Competition and Diversity in Herbaceous Vegetation.” Nature 244: 310. 10.1038/244310a0.

[gcb70521-bib-0036] Ng, J. F. , O. H. Ahmed , M. B. Jalloh , et al. 2022. “Soil Nutrient Retention and pH Buffering Capacity Are Enhanced by Calciprill and Sodium Silicate.” Agronomy 12: 219. 10.3390/agronomy12010219.

[gcb70521-bib-0037] Oksanen, J. , G. L. Simpson , F. G. Blanchet , et al. 2022. “vegan: Community Ecology Package.” R package version 2.6‐4. https://CRAN.R‐project.org/package=vegan.

[gcb70521-bib-0038] Peñuelas, J. , B. Poulter , J. Sardans , et al. 2013. “Human‐Induced Nitrogen–Phosphorus Imbalances Alter Natural and Managed Ecosystems Across the Globe.” Nature Communications 4: 2934. 10.1038/ncomms3934. 24343268

[gcb70521-bib-0039] Peñuelas, J. , J. Sardans , A. Rivas‐ubach , and I. A. Janssens . 2012. “The Human‐Induced Imbalance Between C, N and P in Earth's Life System.” Global Change Biology 18: 3–6. 10.1111/j.1365-2486.2011.02568.x.

[gcb70521-bib-0040] Pérez‐Harguindeguy, N. , S. Díaz , E. Garnier , et al. 2013. “New Handbook for Standardised Measurement of Plant Functional Traits Worldwide.” Australian Journal of Botany 61: 167. 10.1071/BT12225_CO.

[gcb70521-bib-0041] R Core Team . 2022. “R: A Language and Environment for Statistical Computing.” R Foundation for Statistical Computing. Vienna, Austria. https://www.R‐project.org/.

[gcb70521-bib-0042] Rajaniemi, T. K. , V. J. Allison , and D. E. Goldberg . 2003. “Root Competition Can Cause a Decline in Diversity With Increased Productivity.” Journal of Ecology 91: 407–416. 10.1046/j.1365-2745.2003.00768.x.

[gcb70521-bib-0043] Reich, P. B. 2014. “The World‐Wide ‘Fast–Slow’ Plant Economics Spectrum: A Traits Manifesto.” Journal of Ecology 102, no. 2: 275–301. 10.1111/1365-2745.12211.

[gcb70521-bib-0044] Reich, P. B. , S. E. Hobbie , T. D. Lee , and M. A. Pastore . 2018. “Unexpected Reversal of C3 Versus C4 Grass Response to Elevated CO_2_ During a 20‐Year Field Experiment.” Science 360, no. 6386: 317–320. 10.1126/science.aas9313. 29674593

[gcb70521-bib-0045] Revell, L. J. 2012. “Phytools: An R Package for Phylogenetic Comparative Biology (And Other Things).” Methods in Ecology and Evolution 3, no. 2: 217–223. 10.1111/j.2041-210X.2011.00169.x.

[gcb70521-bib-0046] Smil, V. 2000. “Phosphorus in the Environment: Natural Flows and Human Interferences.” Annual Review of Energy and the Environment 25: 53–88. 10.1146/annurev.energy.25.1.53.

[gcb70521-bib-0047] Tälle, M. , B. Deák , P. Poschlod , O. Valkó , L. Westerberg , and P. Milberg . 2016. “Grazing vs. Mowing: A Meta‐Analysis of Biodiversity Benefits for Grassland Management.” Agriculture, Ecosystems & Environment 222: 200–212. 10.1016/j.agee.2016.02.008.

[gcb70521-bib-0048] Tian, Q. , N. Liu , W. Bai , et al. 2016. “A Novel Soil Manganese Mechanism Drives Plant Species Loss With Increased Nitrogen Deposition in a Temperate Steppe.” Ecology 97: 65–74. 10.1890/15-0917.1. 27008776

[gcb70521-bib-0049] Tilman, D. 1982. Resource Competition and Community Structure. Princeton University Press. 7162524

[gcb70521-bib-0050] Tilman, D. , F. Isbell , and J. M. Cowles . 2014. “Biodiversity and Ecosystem Functioning.” Annual Review of Ecology, Evolution, and Systematics 45: 471–493. 10.1146/annurev-ecolsys-120213-091917.

[gcb70521-bib-0051] Tilman, D. , C. L. Lehman , and K. T. Thomson . 1997. “Plant Diversity and Ecosystem Productivity: Theoretical Considerations.” Proceedings of the National Academy of Sciences of the United States of America 94: 1857–1861. 10.1073/pnas.94.5.185. 11038606 PMC20007

[gcb70521-bib-0052] van den Berg, L. J. L. , E. Dorland , P. Vergeer , M. A. C. Hart , R. Bobbink , and J. G. M. Roelofs . 2005. “Decline of Acid‐Sensitive Plant Species in Heathland Can Be Attributed to Ammonium Toxicity in Combination With Low pH.” New Phytologist 166: 551–564. 10.1111/j.1469-8137.2005.01338.x. 15819917

[gcb70521-bib-0053] Violle, C. , and L. Jiang . 2009. “Towards a Trait‐Based Quantification of Species Niche.” Journal of Plant Ecology 2: 87–93. 10.1093/jpe/rtp007.

[gcb70521-bib-0054] Violle, C. , M. Navas , D. Vile , et al. 2007. “Let the Concept of Trait Be Functional!” Oikos 116: 882–892. 10.1111/j.0030-1299.2007.15559.x.

[gcb70521-bib-0055] Wright, I. , P. Reich , M. Westoby , et al. 2004. “The Worldwide Leaf Economics Spectrum.” Nature 428: 821–827. 10.1038/nature02403. 15103368

[gcb70521-bib-0056] Xia, J. , and S. Wan . 2008. “Global Response Patterns of Terrestrial Plant Species to Nitrogen Addition.” New Phytologist 179: 428–439. 10.1111/j.1469-8137.2008.02488.x. 19086179

[gcb70521-bib-0057] Yang, X. , G. Li , S. Li , et al. 2019. “Resource Addition Drives Taxonomic Divergence and Phylogenetic Convergence of Plant Communities.” Journal of Ecology 107, no. 5: 2121–2132. 10.1111/1365-2745.13253.

[gcb70521-bib-0061] Yang, X. , Z. Yang , J. Tan , G. Li , S. Wan , and L. Jiang . 2018. “Nitrogen Fertilization, Not Water Addition, Alters Plant Phylogenetic Community Structure in a Semi‐Arid Steppe.” Journal of Ecology 106: 991–1000. 10.1111/1365-2745.12893.

[gcb70521-bib-0058] Yuan, Z. , S. Jiang , H. Sheng , et al. 2018. “Human Perturbation of the Global Phosphorus Cycle: Changes and Consequences.” Environmental Science & Technology 52: 2438–2450. 10.1021/acs.est.7b03910. 29402084

[gcb70521-bib-0059] Zhang, Z. , G. Wang , H. Wang , Q. Qi , Y. Yang , and J.‐S. He . 2021. “Warming and Drought Increase but Wetness Reduces the Net Sink of CH_4_ in Alpine Meadow on the Tibetan Plateau.” Applied Soil Ecology 167: 104061. 10.1016/j.apsoil.2021.104061.

[gcb70521-bib-0060] Zhu, J. , Y. Zhang , X. Yang , N. Chen , and L. Jiang . 2020. “Synergistic Effects of Nitrogen and CO_2_ Enrichment on Alpine Grassland Biomass and Community Structure.” New Phytologist 228: 1283–1294. 10.1111/nph.16767. 32574402

