# Supplementary material for: Trait‐Mediated Competition for Light Underpins Plant Diversity Loss Under Eutrophication
Source: Glob Chang Biol. 2025 Sep 23;31(9):e70521. doi: 10.1111/gcb.70521 (PMC12455270; doi:10.1111/gcb.70521)
Supplement: Supplementary file 1 — Data S1. [file GCB-31-e70521-s001.pdf]

Supplementary Information

Trait-mediated competition for light underpins plant diversity loss under eutrophication

Tianyuan Tan[1], Huamei Xia[1], Cong He[1], Yao Wei[2], Xiang Liu[1], Zhenhua Zhang[2], Jin-Sheng He[1, 3]*, Lin Jiang[4]*

[1]State Key Laboratory of Herbage Improvement and Grassland Agro-Ecosystems, College of Pastoral Agriculture Science and Technology, Lanzhou University, Lanzhou, China.

[2]Qinghai Haibei National Field Research Station of Alpine Grassland Ecosystems, Northwest Institute of Plateau Biology, Chinese Academy of Sciences, Xining, China.

[3]Institute of Ecology, College of Urban and Environmental Sciences, Key Laboratory for Earth Surface Processes of the Ministry of Education, Peking University, Beijing, China.

[4]School of Biological Sciences, Georgia Institute of Technology, Atlanta, Georgia, USA

Supplementary Table S1. Results of variance inflation factor (VIF) analyses for five plant trait predictors (plant height, SLA, LDMC, LNC, and leaf area) across six generalized linear mixed-effects models (GLMMs). The five traits included were plant height, specific leaf area (SLA), leaf dry matter content (LDMC), leaf nitrogen content (LNC), and leaf area (LA).

Comparison	Type	Height	SLA	LNC	LA	LDMC
**N-Control**	**more losses**	1.63	2.29	1.44	1.24	2.66
**NP-Control**	**more losses**	1.32	2.33	1.36	1.25	2.09
**NL-N**	**more gains**	1.34	2.11	1.33	1.45	1.91
**NPL-NP**	**more gains**	1.29	2.10	1.41	1.25	1.67
**PL-P**	**fewer losses**	1.56	2.37	1.40	1.42	2.72
**NPL-NP**	**fewer losses**	1.29	2.4	1.47	1.25	2.10

Supplementary Table S2. Results of linear mixed-effects models (LMMs) testing the effects of light addition (L), N addition (N) and P addition (P) on soil pH, NO_3^-, NH_4^+, available phosphorus (P), Al^{3+}, Fe^{3+} and Mn^{2+}). Significant results (P <0.05) are indicated in bold.

**Source**	**pH**		**NO_3^- (mg/kg)**		**NH_4^+ (mg/kg)**		**Available P (mg/kg)**	
	**F-value**	***P***	**F-value**	***P***	**F-value**	***P***	**F-value**	***P***
**N**	$0.68_{1,35}$	0.416	$47.34_{1,35}$	**<0.001**	$47.49_{1,35}$	**<0.001**	$8.85_{1,35}$	**0.005**
**P**	$1.89_{1,35}$	0.178	$9.58_{1,35}$	**0.004**	$17.26_{1,35}$	**<0.001**	$43.38_{1,36}$	**<0.001**
**L**	$0.12_{1,35}$	0.737	$0.15_{1,35}$	0.704	$0.074_{1,35}$	0.788	$0.005_{1,35}$	0.945
**N×P**	$0.03_{1,35}$	0.855	$14.56_{1,35}$	**0.001**	$35.1_{1,35}$	**<0.001**	$8.75_{1,36}$	**0.005**
**N×L**	$0.27_{1,35}$	0.610	$2.29_{1,35}$	0.139	$0.06_{1,35}$	0.802	$0.004_{1,35}$	0.948
**P×L**	$0.34_{1,35}$	0.565	$0.13_{1,35}$	0.724	$0.19_{1,35}$	0.666	$0.03_{1,36}$	0.858
**N×P×L**	$0.03_{1,35}$	0.876	$0.01_{1,35}$	0.913	$1.08_{1,35}$	0.305	$0.07_{1,36}$	0.794

**Source**	**Al^{3+} (mg/kg)**		**Fe^{3+} (mg/kg)**		**Mn^{2+} (mg/kg)**	
	**F-value**	***P***	**F-value**	***P***	**F-value**	***P***
**N**	$5.16_{1,35}$	**0.029**	$3.47_{1,35}$	**0.071**	$0.25_{1,35}$	0.618
**P**	$5.45_{1,35}$	**0.025**	$5.86_{1,35}$	**0.021**	$0.48_{1,35}$	0.493
**L**	$1.72_{1,35}$	0.198	$2.33_{1,35}$	0.136	$3.45_{1,35}$	0.072
**N×P**	$1.16_{1,35}$	0.288	$1.09_{1,35}$	0.303	$2.51_{1,35}$	0.122
**N×L**	$0.28_{1,35}$	0.604	$0.23_{1,35}$	0.637	$0.05_{1,35}$	0.823
**P×L**	$1.81_{1,35}$	0.188	$1.76_{1,35}$	0.193	$1.12_{1,35}$	0.298
**N×P×L**	$0.32_{1,35}$	0.573	$0.2_{1,35}$	0.657	$1.68_{1,35}$	0.204

Supplementary Table S3. Results of linear mixed-effects models (LMMs) testing the effects of light addition (L), N addition (N) and P addition (P) on PAR (log-transformed), air temperature and relative humidity. Significant results (*P* <0.05) are indicated in bold.

**Source**	**PAR value (natural)**		**PAR value (natural + added)**		**Air temperature (°C)**		**Relative humidity (%)**	
	**F-value**	***P***	**F-value**	***P***	**F-value**	***P***	**F-value**	***P***
**N**	$193.52_{1,35}$	**<0.001**	$245.33_{1,35}$	**<0.001**	$9.21_{1,35}$	**0.005**	$145.84_{1,35}$	**<0.001**
**P**	$82.81_{1,35}$	**<0.001**	$114.41_{1,35}$	**<0.001**	$9.53_{1,35}$	**0.004**	$39.61_{1,36}$	**<0.001**
**L**	$1.84_{1,35}$	0.18	$213.0_{1,35}$	**<0.001**	$0.342_{1,35}$	0.56	$0.46_{1,35}$	0.500
**N×P**	$26.42_{1,35}$	**<0.001**	$29.32_{1,35}$	**<0.001**	$6.25_{1,35}$	**0.02**	$15.51_{1,36}$	**<0.001**
**N×L**	$0.50_{1,35}$	0.48	$43.77_{1,35}$	**<0.001**	$0.023_{1,35}$	0.88	$0.002_{1,35}$	0.965
**P×L**	$1.46_{1,35}$	0.23	$28.42_{1,35}$	**<0.001**	$0.040_{1,35}$	0.84	$1.41_{1,36}$	0.243
**N×P×L**	$0.28_{1,35}$	0.60	$12.45_{1,35}$	**0.001**	$0.86_{1,35}$	0.36	$0.71_{1,36}$	0.406

Supplementary Table S4. Results of linear mixed-effects models (LMMs) testing the effects of light addition (L), N addition (N) and P addition (P) on community biomass, grass biomass, sedge biomass, legume biomass and forb biomass. Significant results (*P* <0.05) are indicated in bold.

Source	Community biomass		Grass biomass		Forb biomass	
	**F-value**	***P***	**F-value**	***P***	**F-value**	***P***
**N**	$72.46_{1,35}$	**<0.001**	$103.11_{1,40}$	**<0.001**	$1.33_{1,35}$	0.26
**P**	$50.60_{1,35}$	**<0.001**	$68.09_{1,40}$	**<0.001**	$0.86_{1,36}$	0.36
**L**	$0.22_{1,35}$	0.64	$0.78_{1,40}$	0.38	$2.21_{1,35}$	0.15
**N×P**	$12.52_{1,35}$	**0.001**	$26.49_{1,40}$	**<0.001**	$0.10_{1,36}$	0.75
**N×L**	$0.10_{1,35}$	0.75	$0.09_{1,40}$	0.76	$2.76_{1,35}$	0.11
**P×L**	$0.73_{1,35}$	0.40	$1.19_{1,40}$	0.28	$0.04_{1,36}$	0.84
**N×P×L**	$1.90_{1,35}$	0.18	$2.33_{1,40}$	0.13	$0.00_{1,36}$	0.94

Source	Legume biomass		Sedge biomass	
	**F-value**	***P***	**F-value**	***P***
**N**	$65.88_{1,35}$	**<0.001**	$21.43_{1,35}$	**<0.001**
**P**	$6.39_{1,35}$	**0.02**	$59.05_{1,35}$	**<0.001**
**L**	$0.10_{1,35}$	0.75	$0.31_{1,35}$	0.58
**N×P**	$12.26_{1,35}$	**0.001**	$22.78_{1,35}$	**<0.001**
**N×L**	$0.00_{1,35}$	0.95	$0.09_{1,35}$	0.77
**P×L**	$0.00_{1,35}$	0.96	$2.81_{1,35}$	0.10
**N×P×L**	$0.03_{1,35}$	0.86	$1.16_{1,35}$	0.29

Supplementary Table S5. Results of linear mixed-effects models (LMMs) testing the effects of light addition (L), N addition (N) and P addition (P) on species richness (log-transformed), and the number of species losses and gains (log-transformed). Significant results (*P* <0.05) are indicated in bold.

**Source**	**Richness**		**Species losses**		**Species gains**	
	**F-value**	***P***	**F-value**	***P***	**F-value**	***P***
**N**	$44.54_{1,35}$	**<0.001**	$61.68_{1,35}$	**<0.001**	$6.80_{1,40}$	**0.01**
**P**	$0.96_{1,35}$	0.34	$3.27_{1,35}$	0.08	$0.38_{1,40}$	0.54
**L**	$21.40_{1,35}$	**<0.001**	$17.63_{1,35}$	**<0.001**	$4.59_{1,40}$	**0.04**
**N×P**	$8.31_{1,35}$	**0.01**	$10.39_{1,35}$	**<0.01**	$2.09_{1,40}$	0.16
**N×L**	$9.15_{1,35}$	**<0.01**	$1.33_{1,35}$	0.26	$7.02_{1,40}$	**0.01**
**P×L**	$3.69_{1,35}$	0.06	$5.83_{1,35}$	**0.02**	$1.88_{1,40}$	0.18
**N×P×L**	$0.73_{1,35}$	0.40	$1.97_{1,35}$	0.17	$1.52_{1,40}$	0.22

Supplementary Table S6. Results of generalized linear mixed-effects models (GLMMs) for treatment-induced species losses and gains as a function of height, SLA, leaf nitrogen content, leaf area and LDMC. The optimal model is retained for each analysis. For each model, the degrees of freedom (df), maximum log-likelihood (LogLik), information-theoretic Akaike's information criterion corrected for small samples (AICc) are provided as measures of model goodness-of-fit. Significant levels: ^*P*<0.1, **P*<0.05, and ***P*<0.01.

Comparison	Type	Intercept	Height	LDMC	Leaf area	Leaf N	SLA	df	logLik	AICc
**N-Control**	**more losses**	-2.12		-0.68**				3	-83.67	173.5
**NP-Control**	**more losses**	-1.63		-0.74***	-0.61*			4	-147.55	303.2
**NL-N**	**more gains**	-2.60	-0.62^		0.28			4	-62.98	134.1
**NPL-NP**	**more gains**	-2.70	-0.97**					3	-86.6	179.2
**PL-P**	**fewer losses**	-2.48	0.43	-0.93**				4	-84.58	177.3
**NPL-NP**	**fewer losses**	-2.00	-0.38^				0.25	4	-120.56	249.2

Supplementary Table S7. Phylogenetic signal (Blomberg's *K*) of greater versus fewer gains or losses between treatments

Comparison	Type	Blomberg's *K*	*P* value
**N-Control**	**more losses**	0.11	0.51
**NP-Control**	**more losses**	0.07	0.69
**NL-N**	**more gains**	0.21	0.06
**NPL-NP**	**more gains**	0.10	0.48
**PL-P**	**fewer losses**	0.10	0.41
**NPL-NP**	**fewer losses**	0.11	0.39

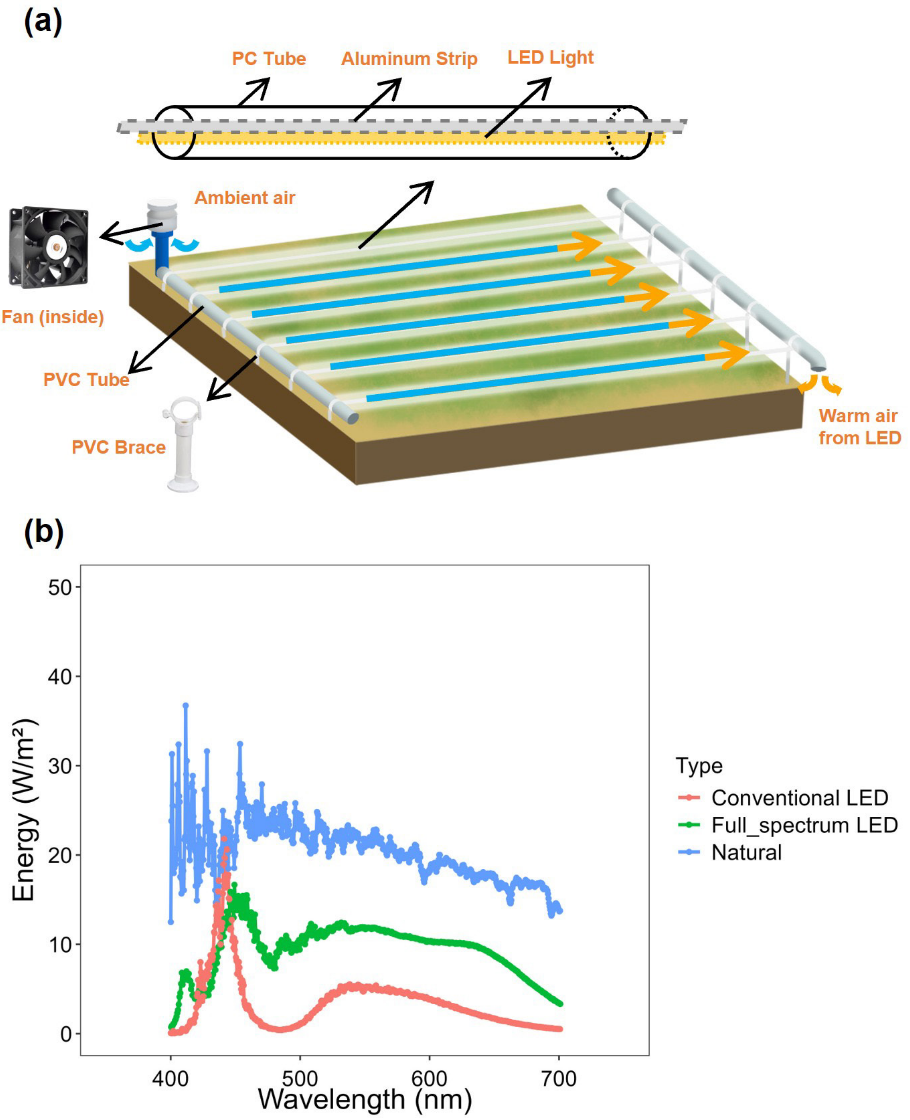

Supplementary Figure S1. Understory light addition device used in the field experiment and spectral characteristics of LED light. (a) light addition device. The device consists of LED strips, a circulating fan, PVC/PC tubes, PVC braces, and aluminum strips (preventing light exposure in the no-light treatments). The circulating fan directs ambient air into PVC tubes, effectively carrying away the heat emitted by the LED strips. (b) Spectral profiles of three light sources: natural sunlight, full-spectrum LED strips, and conventional LED strips. For natural sunlight, a measurement from a clear day at a single time point was used for comparison.

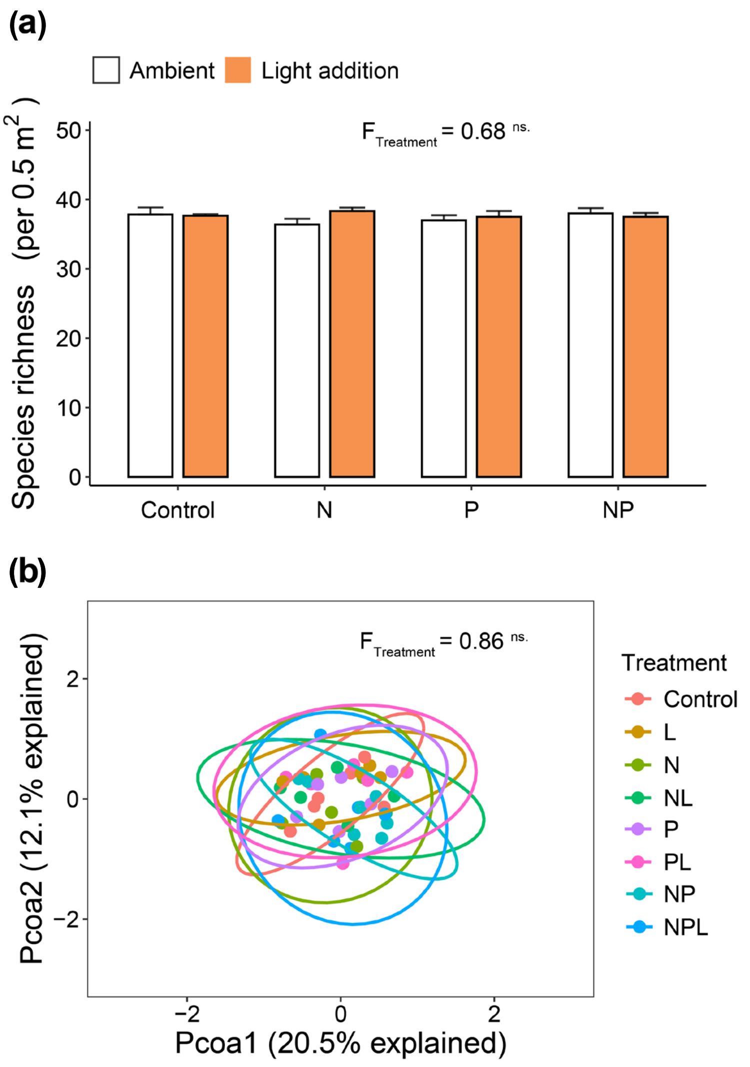

Supplementary Figure S2. Initial richness and community structure in 2020. Treatment effects on (a) species richness and (b) community composition in the initial year (2020). Species richness was analyzed using one-way ANOVA, and community composition was assessed by PCoA of Bray–Curtis dissimilarities with PERMANOVA. No significant treatment effects were detected in either case (ns.). Data are presented as mean ± s.e.m. for species richness.

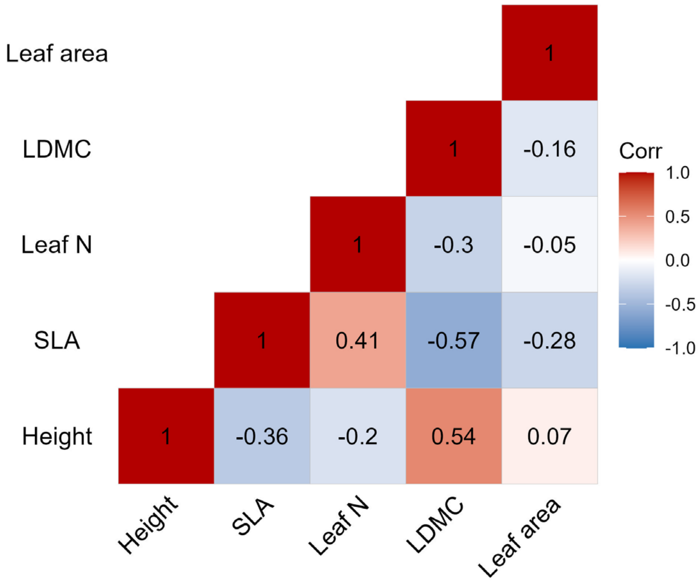

Supplementary Figure S3. Pearson correlation coefficients among the five plant traits: plant height, specific leaf area (SLA), leaf dry matter content (LDMC), leaf nitrogen content, and leaf area; all |r| < 0.6, suggesting limited collinearity among traits.

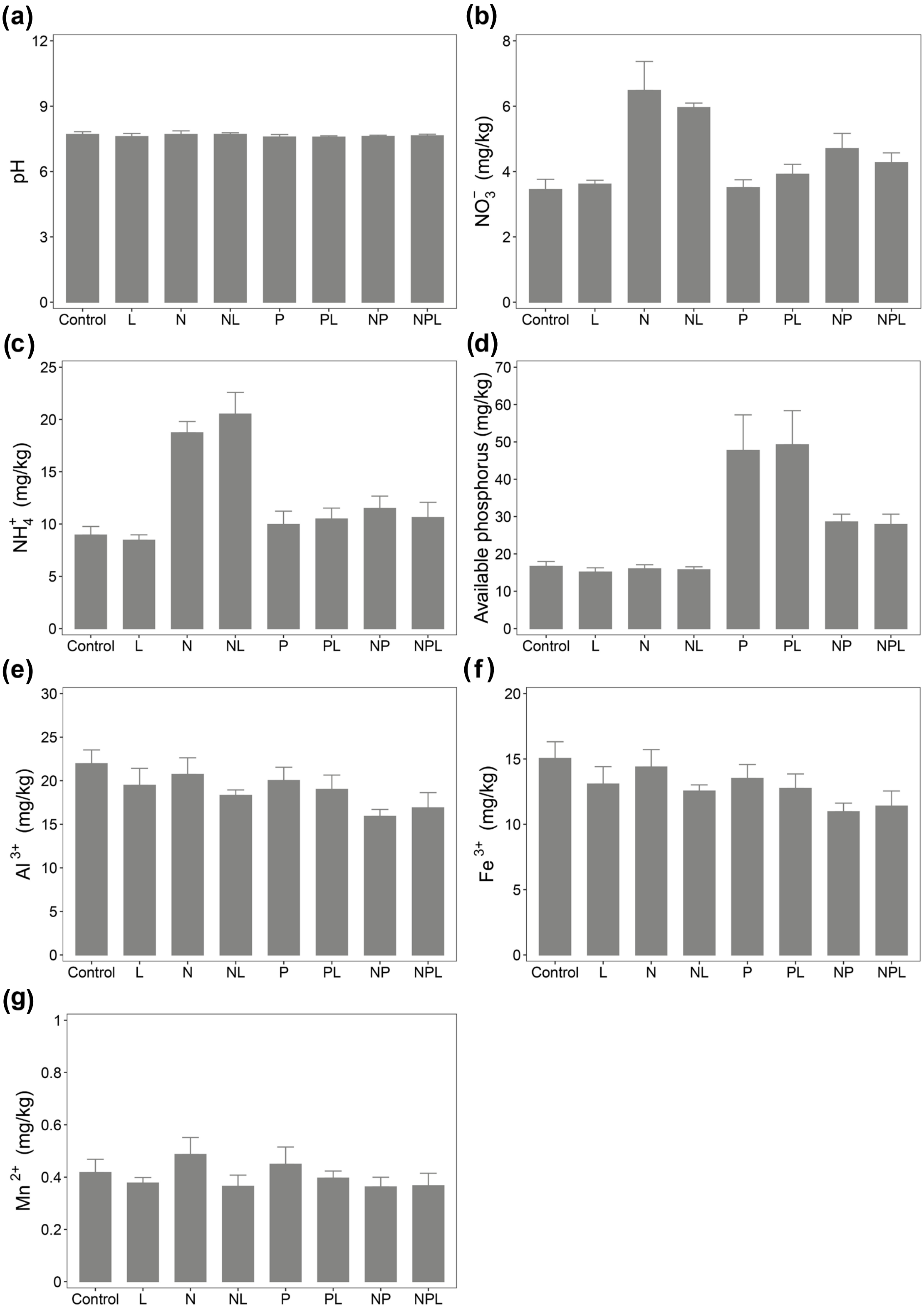

Supplementary Figure S4. Effects of experimental treatments on soil properties. The effects of light addition (L), N addition (N) and P addition (P) on soil properties (**a**: pH, **b**: NO_3^-, **c**: NH_4^+, **d**: available phosphorus, **e**: Al^{3+}, **f**: Fe^{3+}, and **g**: Mn^{2+}). Data are presented as mean + s.e.m.

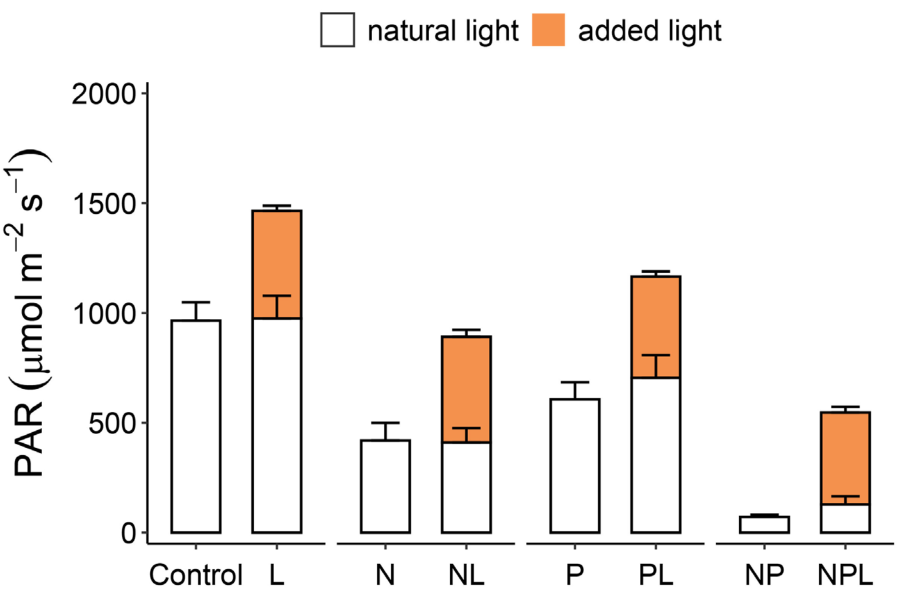

Supplementary Figure S5. Effects of experimental treatments on photosynthetically active radiation (PAR). The plot shows the effects of light addition (L), N addition (N) and P addition (P) on PAR levels in the understory. Natural and added light intensity are shown in white and orange colors, respectively. PAR measurement was taken at 10 cm aboveground. Data are presented as mean + s.e.m.

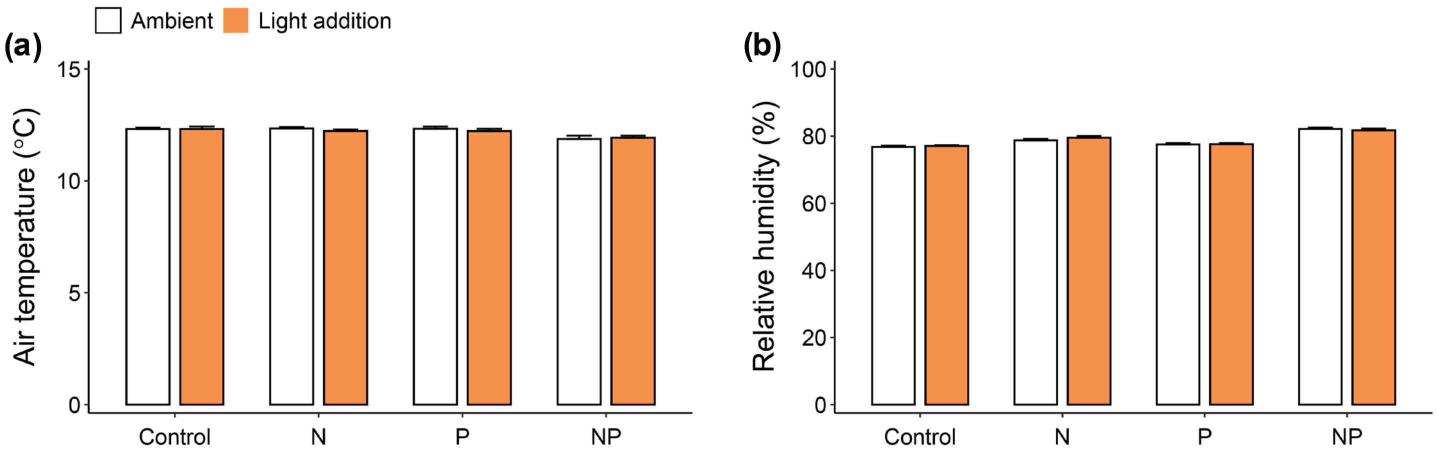

Supplementary Figure S6. Effects of experimental treatments on microclimate. The effects of light addition (L), N addition (N) and P addition (P) on air temperature (**a**) and relative humidity (**b**) in the experimental plots. Data are presented as mean + s.e.m.

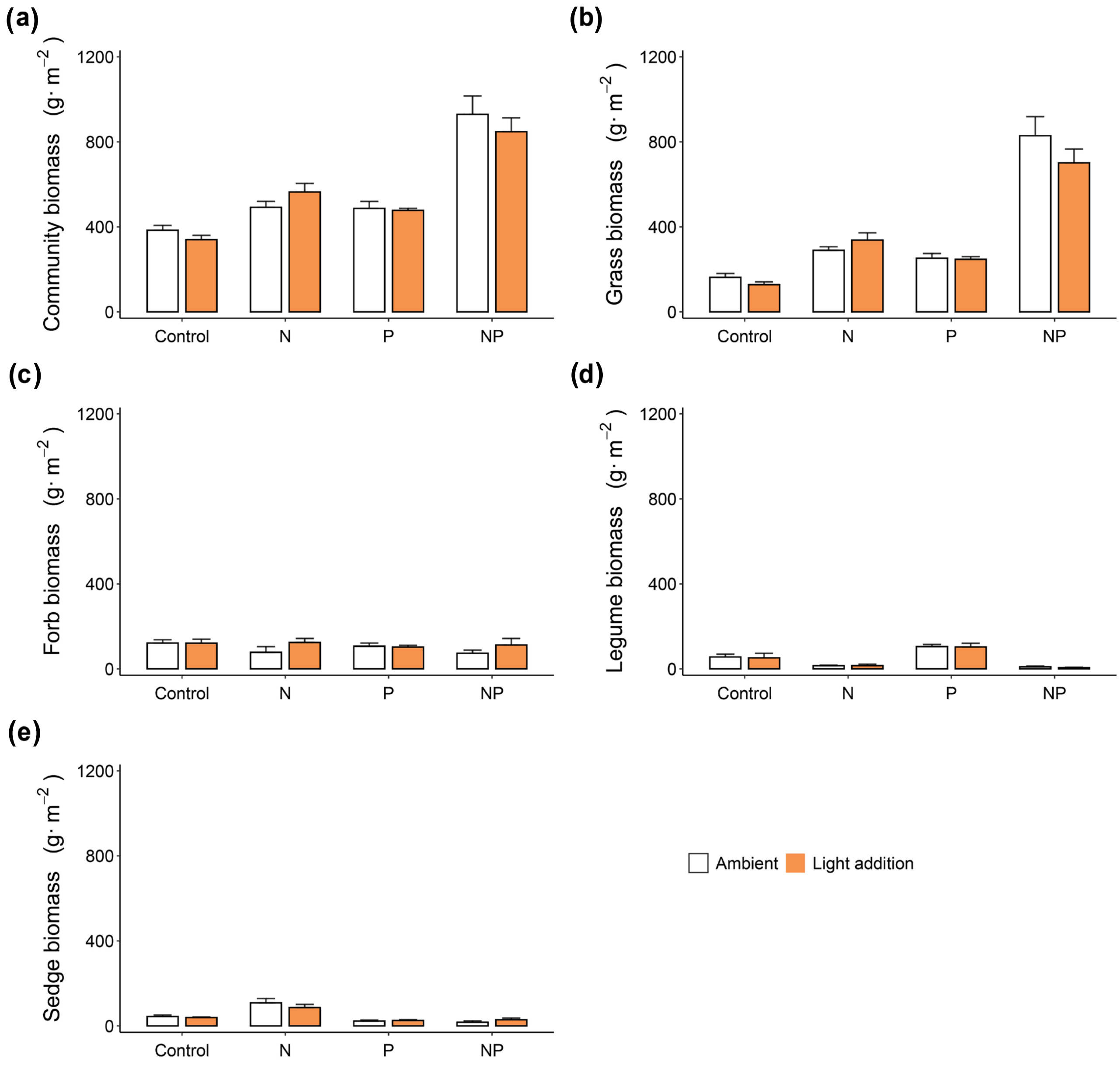

Supplementary Figure S7. Effects of experimental treatments on plant biomass. The effects of light addition (L), N addition (N) and P addition (P) on total community biomass (**a**), grass biomass (**b**), forb biomass (**c**), sedge biomass (**d**), and legume biomass (**e**). Data are presented as mean + s.e.m.

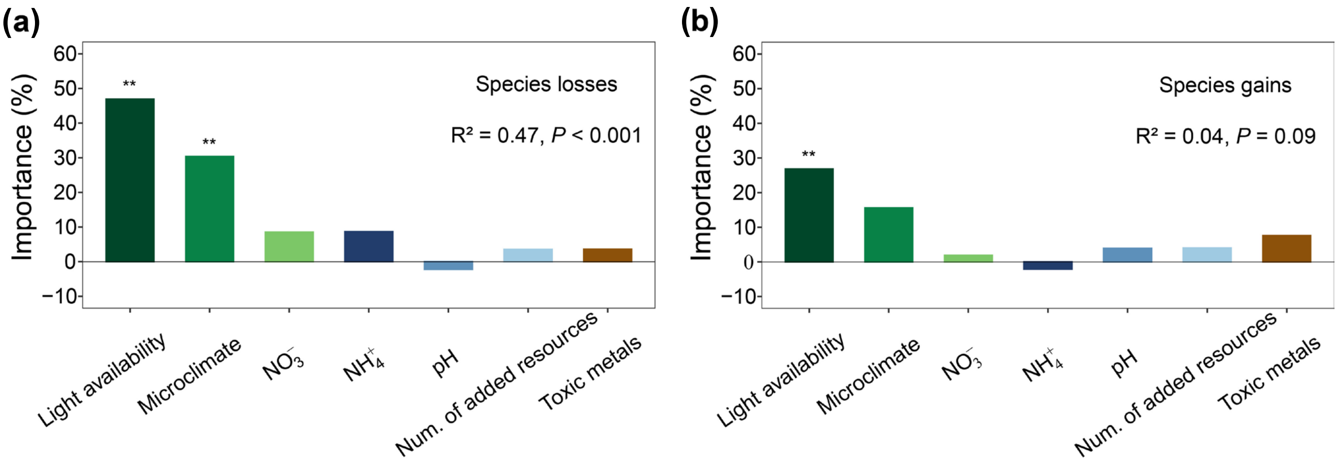

Supplementary Figure S8. The results of the random forest model for predictors of species losses and gains. Random Forest mean predictor importance of light availability, niche dimension (i.e., the number of added resources), soil toxicity metals, as well as microclimate, for species losses (**a**) and gains (**b**). Significant levels: **P* < 0.05 and ***P* < 0.01.

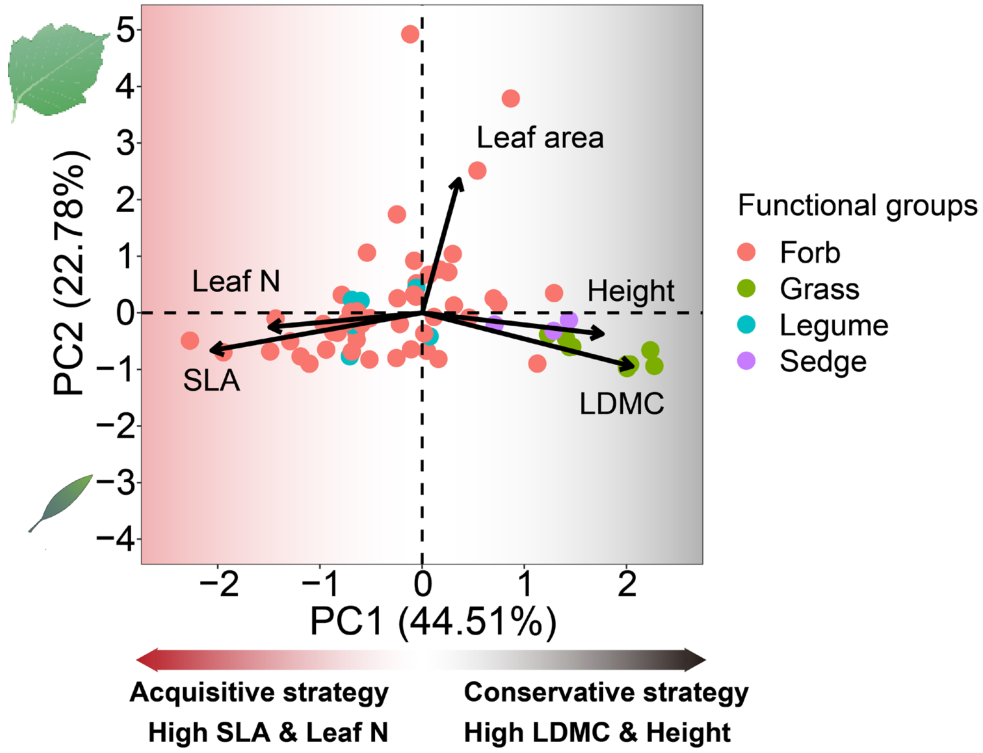

Supplementary Figure S9. Principal component analyses (PCA) of the five functional traits (height, leaf N, SLA, LDMC and leaf area) of all 68 species present in the experimental plots. The PCA highlights the growth strategies of the four functional groups: grasses, forbs, legumes, and sedges. Axis labels show the percentage of variation explained by each principal component. Leaf graphics by T. Saxby and L. Heydon (Integration and Application Network, University of Maryland Center for Environmental Science, http://ian.umces.edu/imagelibrary/).
